# Fighting the enemy: one health approach against microbial resistance

**DOI:** 10.1111/1751-7915.13587

**Published:** 2020-05-04

**Authors:** Zulema Udaondo, María José Huertas

**Affiliations:** ^1^ Department of Biomedical Informatics University of Arkansas for Medical Sciences Little Rock AR 72205 USA; ^2^ Instituto de Bioquímica Vegetal y Fotosíntesis Universidad de Sevilla‐CSIC Américo Vespucio 49 41092 Sevilla Spain

## Abstract

This article highlights publications in Enviromental Microbiology and Microbial Biotechnology papers about antibiotic resistance. It concludes that the One health approach is basic to addressing this problem.

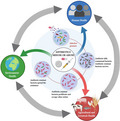

Penicillin wide availability in the mid‐twentieth century marked the beginning of the antibiotic era. Since then, antibiotic treatments have saved millions of lives, revolutionizing the treatment of infectious diseases worldwide and increasing our life expectancy (Davies and Davies, 2010). However, the extensive use of antimicrobial drugs has triggered what could be one of the biggest threats to human, animal and environmental health in the 21st century: antimicrobial resistance (AMR) (WHO, 2015). In fact, some predictions estimate that deaths attributable to AMR may rise from 700 000 to 10 million lives annually by 2050 (Brogan and Mossialos, 2016; Butler, *et al.*, 2017).

Infections caused by multidrug‐resistant (MDR) strains were first reported in hospital settings, where antimicrobial use was most prevalent. Nevertheless, nowadays antibiotic compounds are essential not only for human therapy but also for prophylaxis and growth promotion in the animal food industry (Castanon, 2007). The exposure to substantial quantities of antimicrobial compounds in farm animals has made livestock products a potential reservoir of antimicrobial‐resistant genes (ARGs), raising the chances of transmission to humans through the food chain, by direct animal contact, and through the environment (e.g. wastewater), contributing to the spread of antibiotic resistance (Maestre‐Carballa *et al.*, 2019).

Evidence of the dissemination of MDR clones and mobile genetic clusters by antibiotic resistance cassettes via food chain from poultry to humans is presented by Cohen and colleagues in a comprehensive paper in *Environmental Microbiology* using 16 438 poultry and 27 489 clinical (human) *Salmonella* isolates (Cohen *et al.*, 2020). The study by Cohen *et al.* illuminates the ecology and resistance mechanisms of 13 common poultry‐associated *Salmonella* serovars demonstrating a high MDR prevalence in a subset of the strains. This work emphasizes the role of the poultry production industry as a reservoir of epidemic MDR strains and mobile genetic elements that confer resistance to medically relevant antibiotics.

**Fig. 1:**
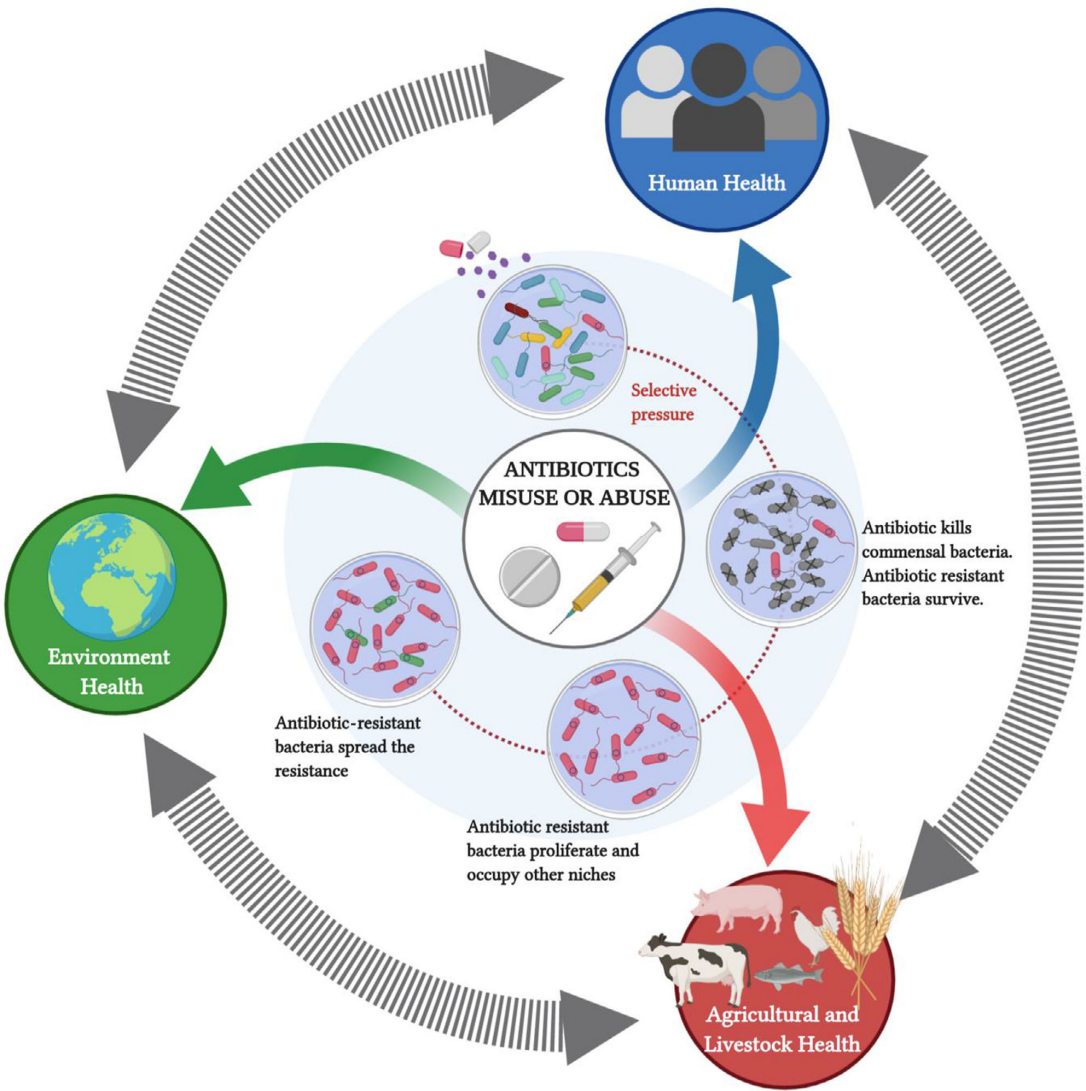
The One Health approach holds that the health of human beings is connected to the health of animals, plants and our shared environment. The inner‐circle illustrates how antibiotic resistance is spread in a healthy and diverse bacterial population. This figure was made using Biorender

One of the first natural barriers in the fight against the infection by antibiotic‐resistant bacteria is the intestinal microbiota. The gut microbiome is critical for homeostasis in humans and animals (Clemente *et al.*, 2012); however, the use of broad‐spectrum antibiotics causes a general imbalance of the resident flora (dysbiosis) that can potentially have consequences for animal and human health (Clemente *et al.*, 2012; Cully, 2019). One of these consequences is a disproportional increase in the abundance of specific bacteria that may carry mobile elements containing ARGs. The study by Dobrzanska and colleagues (2020) in *Microbial Biotechnology* correlates changes in gut microbiota composition, after preventive antibiotic therapy, with weight gain and disease in calves. In this work, calves treated with Florfenicol changed their gut microbiota composition with enrichment of a facultative anaerobe Proteobacteria (mainly *E. coli*) and consequently of *E. coli* mobile‐mediated ARGs that provided resistance to other drugs not administered to the calves. Antibiotic treated calves also showed an increase in body weight compared to the non‐treated group, although both groups followed the same diet. Authors propose that antibiotic treatment of healthy animals led to unbalanced, disease‐ and obese‐related microbiota, that promotes the growth of microorganisms carrying clinically relevant mobile ARGs, and potentially increasing the risk of transmission of antibiotic‐resistant bacteria to humans (Dobrzanska *et al.*, 2020).

The use of antibiotics as a growth promoter agent has been deemed problematic due to multiple reasons (Silbergeld *et al.*, 2008; Verraes *et al.*, 2013). One of the most important consequences is the fate of all those administered antibiotics that are not absorbed by the animal and dumped into the environment in the way of faeces and urine. This situation, along with the insufficient treatment of residues from industrial, residential and farm wastes, contributes to the expansion of the environmental resistome (Chee‐Sanford *et al.*, 2009; Del Fiol *et al.*, 2018). This resistome is in part due to the influx of ARM genes from environmental bacteria that can be incorporated through horizontal gene transfer (HGT) into commensals and pathogenic strains. A recent study by Mourkas and colleagues in *Environmental Microbiology* addresses this topic, providing a basis for understanding the interaction between different AMR gene pools and the potential source/sink contribution of livestock, humans and sewage effluents in the *Campylobacter* resistome. In their report, the authors highlight the existence of multiple HGT events within *Campylobacter* strains from the gut microbiome, evidenced by the existence of genetic islands (GIs) that contain clusters of genes for resistance to multiple drug classes and that are shared among isolates from multiple sources (livestock, humans and sewage) (Mourkas, *et al.*, 2019).

Given that the effectiveness of an antimicrobial compound is determined by the possible tolerance or resistance of the targeted bacteria, understanding the drivers of antibiotic resistance and the mechanisms of selection for resistant bacteria is of vital importance. Mechanisms of antibiotic resistance have been well documented for several drug classes. In a nutshell, these mechanisms block drug function by reducing its intracellular concentration via efflux pumps, reducing the membrane permeability, or modifying the antimicrobial compound or its target in the cell (Munita and Arias, 2016). However, there is still a lack of knowledge in certain antibiotic resistance mechanisms being necessary the devise of innovative therapeutic approaches. Comparative metabolomics analysis between sensitive and resistant strains from the same species could help to break ground on new approaches to eliminate MDR bacteria. In connection with the latter, the work by Zhang and coworkers revealed for the first time an unknown metabolic mechanism in *Vibrio alginolyticus* for gentamicin resistance. The authors show that gentamicin resistant strains of *Vibrio* have mutations on genes related to carbon and energy metabolism indicating a metabolic shift. Mutations that produce the gentamicin resistance phenotype affect the energy production of the cell, leading to altered operation of metabolic pathways such as the pyruvate cycle and the sodium‐pumping NADH: ubiquinone oxidoreductase (Na(+)‐NQR) system, which in turn decreased the membrane potential and a concomitant decrease in the intracellular concentration of gentamicin (Zhang *et al.*, 2019). Further analysis of the metabolic profile of gentamycin resistant *V. alginolyticus* strains identified glucose as the most down‐regulated metabolite, and the authors proposed that glucose in this microbe can function as an antibiotic resistance‐reverting molecule.

The use of alternative therapies to broad‐spectrum antibiotics such as bacteriocins, CRISPR‐Cas antimicrobials, bacteriophage therapy, the local release of toxins, and the use of predatory bacteria have been reported in recent years (Kadouri *et al.*, 2013; Allen, 2017; Ghosh *et al.*, 2019). Unfortunately, none of these options have consistently demonstrated efficacy comparable to antibiotic treatments. Given the important interactions between the different contributions of human, animal and environmental sources to antimicrobial resistance, it is logical to take into consideration a One Health perspective when addressing this problem (McEwen and Collignon, 2018). The One Health approach recognizes that humans, animals and environmental health are intricately linked, and, therefore, the control and surveillance of ARG needs to be regarded in all three sectors (Fig. 1). The complexities of mechanisms by which microorganisms can acquire ARGs are driven not only by biological processes but it is also affected by socio‐economic factors that should be broken down into its components for their study, and they should be taken into account to design of strategies to combat this public health threat. These factors contribute to genetic selection pressure for the emergence of MDR bacterial infections in the community. Integrated measures of inspection, surveillance, next‐generation sequencing and advanced bioinformatics tools, while implementing the One Health approach, are required to better control antibiotic usage in animal production, and to reduce contamination and transmission of MDR strains along the food chain.

## Conflict of interest

None declared.
